# Detection of Methylated *CDO1* in Plasma of Colorectal Cancer; A PCR Study

**DOI:** 10.1371/journal.pone.0113546

**Published:** 2014-12-03

**Authors:** Keishi Yamashita, Mina Waraya, Myoung Sook Kim, David Sidransky, Natsuya Katada, Takeo Sato, Takatoshi Nakamura, Masahiko Watanabe

**Affiliations:** 1 Department of Surgery, Kitasato University Hospital, 1-15-1 Kitasato, Minami-ku, Sagamihara, Kanagawa, 252-0375, Japan; 2 Department of Otolaryngology, Head and Neck Cancer Research Institute, Johns Hopkins University, 1550 Orleans Street, Baltimore, Maryland, 21231, United States of America; University of Medicine, Greifswald, Germany

## Abstract

**Background:**

Cysteine biology is important for the chemosensitivity of cancer cells. Our research has focused on the epigenetic silencing of cysteine dioxygenase type 1 (CDO1) in colorectal cancer (CRC). In this study, we describe detection of *CDO1* methylation in the plasma of CRC patients using methylation specific PCR (Q-MSP) and extensive analysis of the PCR reaction.

**Methods:**

DNA was extracted from plasma, and analysed for methylation of the *CDO1* gene using Q-MSP. The detection rate of *CDO1* gene methylation was calculated and compared with that of diluted DNA extracted from “positive control” DLD1 cells. *CDO1* gene methylation in the plasma of 40 CRC patients that were clinicopathologically analysed was then determined.

**Results:**

(**1**) The cloned sequence analysis detected 93.3% methylation of the promoter CpG islands of the *CDO1* gene of positive control DLD1 cells and 4.7% methylation of the negative control HepG2 *CDO1* gene. (**2**) DLD1 *CDO1* DNA could not be detected in this assay if the extracted DNA was diluted ∼1000 fold. The more DNA that was used for the PCR reaction, the more effectively it was amplified in Q-MSP. (**3**) By increasing the amount of DNA used, methylated *CDO1* could be clearly detected in the plasma of 8 (20%) of the CRC patients. However, the percentage of CRC patients detected by methylated *CDO1* in plasma was lower than that detected by CEA (35.9%) or CA19-9 (23.1%) in preoperative serum. Combination of CEA/CA19-9 plus plasma methylated *CDO1* could increase the rate of detection of curable CRC patients (39.3%) as compared to CEA/CA19-9 (25%).

**Conclusion:**

We have described detection of *CDO1* methylation in the plasma of CRC patients. Although *CDO1* methylation was not detected as frequently as conventional tumor markers, analysis of plasma *CDO1* methylation in combination with CEA/CA19-9 levels increases the detection rate of curable CRC patients.

## Introduction

Cytosine DNA methylation of the promoter region of tumor suppressor genes is a common cancer phenomenon [1], [2], and could provide good candidate biomarkers for detection of minimal residual disease using methylation specific PCR amplification [3], [4]. DNA methylation alterations differ from genomic alterations such as mutations in that methylation abnormalities occur frequently enough for application to clinical diagnosis [5]. For example, we have identified *N-methyl-D-aspartate receptor type 2A* (*NMDAR2A*) [6], *deafness, autosomal dominant 5* (*DFNA5*) [7], *Oncostatin M receptor-*β (*OSMR*) [8], and *cysteine dioxygenase 1* (*CDO1*) [9] as cancer-prone frequently methylated genes in colorectal cancer (CRC) by using pharmacological unmasking microarrays [1], [2]. Of these genes, the cancer-prone genes that was most frequently methylated in primary CRC was *CDO1* [9].

Cysteine biology has recently gained attention in terms of tumor biology because it involves reactive oxygen (ROS) production and influences the chemosensitivity of cancer cells through the CD44-xCT (cancer stem cell marker-cysteine transporter) axis [10], [11]. CDO1 affects cytosine metabolism, resulting in the generation of reactive oxygen species (ROS) and the reduction of cell viability and growth [12], [13]. Hence, *CDO1* is believed to be a critical tumor suppressor gene, and could be a marker of the chemo-resistance of cancer cells.

Since DNA methylation was first detected in the plasma of esophageal cancer patients [14], methylated DNA has been repeatedly found in the plasma of various cancers including CRC [15]. *SEPT9* promoter was considered to be the most promising tumor marker, because 69% of the primary CRC tissues were positive for this methylation, whereas it was not detected in 86% of the controls. Further validation studies proved that a plasma *SEPT9* methylation assay could detect CRC with high sensitivity [16], [17].

In the present study, we addressed the outstanding *CDO1* hypermethylation that was observed in primary CRC tissues: 91% of the tumor tissues were positive for *CDO1* methylation, whereas it was not detected in 93% of the controls [9]. We describe detection of CDO1 methylation in the plasma of CRC patients and extensive analysis of the PCR reaction.

## Methods

### Cell lines and plasma samples

The CRC cell line DLD1 was kindly provided by the Cell Resource Centre for Biomedical Research, Institute of Development, Aging and Cancer, Tohoku University (Sendai, Japan). The hepatocellular carcinoma cell line HepG2 was purchased from the RIKEN BioResource Centre (Ibaraki, Japan). DLD1 cells were grown in RPMI 1640 medium (GIBCO, Carlsbad, CA) containing 10% fetal bovine serum (FBS). HepG2 cells were grown in DMEM medium (GIBCO), supplemented with 10% FBS.

Plasma from 20 (for the preliminary study) and 40 (for the validation study) patients with primary CRC was collected before the patients underwent surgical resection at the Kitasato University Hospital from September 1, 2009 to June 30, 2011. The present study was approved by the Ethics Committee of Kitasato University. Participants provided their written informed consent to participation in this study prior to surgery.

CRC stage was categorized according to the TNM classification, 7^th^ edition of the Union for International Cancer Control (UICC) and the 7^th^ edition of the Japanese Classification of Colorectal Cancer (JCCC) staging system. Patient characteristics are shown in Table 1.

**Table 1:** Methylated *CDO1* in plamsa and clinicopathological features in CRC.

Clinicopathological factors	Patient Number	Methylated *CDO1* in plasma		P value
		Present (n = 9)	Absent (n = 31)	
Age of years				NS
equal to or over 60	30	6	24	
below 60	10	3	7	
Sex				NS
Male	21	5	16	
Female	19	4	15	
Tumor location				NS
Right colon	21	6	15	
Left colon	15	2	13	
Rectum	4	1	3	
T factor				NS
pT1	2	0	2	
pT2	6	1	5	
pT3	19	6	13	
pT4	13	2	11	
N factor				NS
pN0	17	3	14	
pN1	8	1	7	
pN2/N3/NX	16	5	11	
M factor				NS
pM0	29	5	24	
pM1	11	4	7	
Preoperative serum CEA				NS
High value (> or = 5 ng/ml)	14	3	11	
Low value (<5 ng/ml)	25	6	19	
Preoperative serum CA19-9				NS
High value (> or = 37 IU/ml)	9	3	6	
Low value (<37 IU/ml)	30	6	24	

NS, not significant.

### DNA extraction

Free floating circulating DNA was extracted from plasma using the MagNA Pure Compact Nucleic Acid Isolation kit I (Roche Applied Science, Mannhein, Germany) and a Roche MagNA Pure device. Plasma (400 µL: small amounts, and 1 to 2.5 ml: large amounts) were distributed in MagNA Pure wells and were, extracted following the kit protocol. Genomic DNA was eluted in 100 uL aliquots. And genomic DNA from cell lines was extracted using the QIAamp DNA Mini Kit (QIAGEN Sciences, Hilden).

### Bisulfite Treatment of DNA and Sequencing Analysis

For DNA denaturing, DNA extracted from the plasma of CRC patients (final volume 100 µL) or 2 µg of genomic DNA obtained from cell lines, were incubated with 5 µg of salmon sperm DNA in 0.3 mol/l NaOH for 20 min at 50°C. The DNA sample was then diluted with 500 µl of 2.5 mol/l sodium metabisulfite (Sigma-Aldrich Inc., St. Louis, MO)/125 mmol/l hydroquinone (Sigma)/0.4 mol/l sodium hydroxide solution, and placed at 70°C for 1.5 hours. The sample was subsequently applied to a column (Wizard DNA Clean-UP System, Promega Inc., Madison, WI), incubated with 0.3 mol/l NaOH for 10 min, and then treated with 3 mol/l ammonium acetate for 5 min. The sample was precipitated in 100% ethanol, and the DNA was resuspended in 25 or 50 µl of LoTE composed of 10 µM Tris-HCl, pH 8 and 2.5 µM ethylenediaminetetraacetic acid (EDTA), pH 8. The DNA from cell lines was subsequently amplified using a polymerase chain reaction (PCR). Bisulfite treatment results in the chemical modification of unmethylated, but not methylated, cytosines to uracils, allowing the distinction between methylated and unmethylated genomic DNA. Primer sequences for the genes of interest were designed to recognize these DNA alterations (Table S1). The primer products were sequenced using a Big Dye Terminator v3.1 Cycle Sequencing Kit (Applied Biosystems, Foster City, CA).

For analysis of the cloned sequences, the PCR products were inserted into the pCR4-TOPO vector using a TOPO TA cloning kit for sequencing (Invitrogen, Carlsbad, CA, USA). Ten clones were selected for each sample, which were then sequenced using semi-nested primers (Table S1).

### Quantitative-Methylation-specific PCR (Q-MSP)

TaqMan methylation specific PCR (Q-MSP) was carried out in triplicate using the iQ Supermix (Bio-Rad), and the iCycler iQ Real-Time PCR Detection system (Bio-Rad). PCR conditions and the primer sequences are provided in Table S1. Serial dilutions of bisulfite modified DNA from DLD1 were used as the methylation positive control to construct a calibration curve on each plate, and modified DNA from HepG2 cells was used as negative control.

### Statistical Analysis

Categorical variables were evaluated by Fisher's exact test. Survival was calculated by the Kaplan–Meier method. Univariate analyses of prognostic factors for progression free survival (PFS) were performed using the log-rank method. PFS was defined as time from surgery to progression (recurrence or R1/2 operation) or deaths from any causes, and data on surviving patients were censored at the last follow-up. The median follow-up was 46 months (range: 0–55 months).

## Results

### Promoter DNA hypermethylation of the *CDO1* gene by analysis of cloned sequence from human cancer cell lines for establishment of positive and negative controls for quantitative methylation analysis

**Figure 1:**
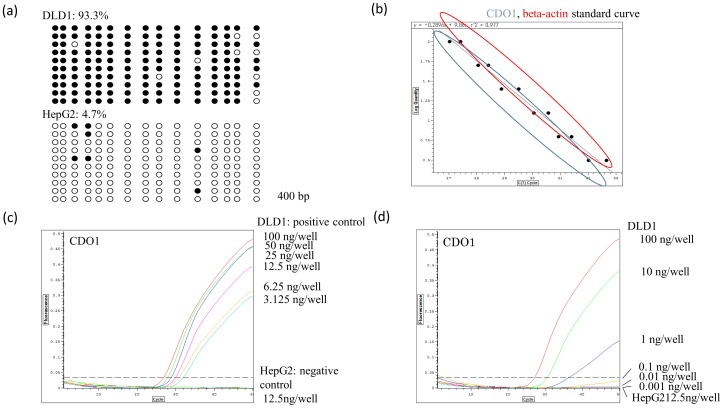
Q-MSP analysis of *CDO1* methylation and analysis of the PCR reaction. (**a**) Sequence analysis of the cloned *CDO1* promoter. The frequency of methylated CpG islands in the cloned *CDO1* promoter from the positive control, DLD1 cells, and the negative control, HepG2 cells. (**b**) Q-MSP efficacy for PCR amplification of *CDO1* and β-actin was excellent. (**c**) Sensitivity of *CDO1* methylation detection using Q-MSP was assayed using 2-fold dilutions (from 100 to 3.125 ng/PCR) of cloned *CDO1* promoters from DLD1 and HepG2 cells. (**d**) Q-MSP of *CDO1* methylation in in DLD1 cells using 10-fold dilutions (100, 10, 1, 0.1, and 0.01 ng/PCR) of the cloned *CDO1* promoter. A concentration as low as 1 ng/PCR reaction of the positive control DLD1 cell DNA can be clearly detected. However, DNA at 1000 fold dilution (0.1 ng/PCR) can be barely be detected. Note that detection is less sensitive when lower amounts of DNA are applied.

Although *CDO1* is clearly hypermethylated in cancer cell lines [9], the degree of *CDO1* promoter hypermethylation of cloned *CDO1* promoter sequences has not been determined. Using bisulfite treatment of DNA followed by sequence analysis, we analyzed the methylation level of the *CDO1* promoter sequence cloned from PCR amplified genomic DNA of DLD1 (methylation positive) and HepG2 (methylation negative) cells. This assay indicated methylation of 93% and 4.7% of the CpG sites of the *CDO1* promoter region in DLD1 and HepG2 cells, respectively (Fig. 1a). We therefore considered that DLD1 and HepG2 cell lines were appropriate for use as positive and negative controls, respectively, for *CDO1* methylation analysis.

### Q-MSP of the methylated CpG sites of *CDO1* gene clearly amplified a specific signal in the positive control, DLD1, but not in the negative control, HepG2

We next quantified methylated *CDO1* after bisulfite DNA treatment using Q-MSP with the same set of primers and probe that was previously reported [9]. Using this method, amplified signals could be clearly identified. The efficacy of PCR amplification of methylated *CDO1* was excellent and was found to be comparable with β-actin amplification efficacy (Fig. 1b). Q-MSP of *CDO1* methyaltion indicated that methylation could be clearly detected when the DNA template from DLD1 cells was used at concentrations of 100, 50, 25, 12.5, 6.25, and 3.125 ng/PCR reaction, whereas no methylation at all could be detected in negative control HepG2 DNA templates (Fig. 1c). We used fluorescence of 0.35 as the threshold line in this assay, because the negative control is always below this threshold. When the DNA template was further diluted concentrations of 100, 10, 1, 0.1, and 0.01 ng/PCR reaction were analyzed, Q-MSP of *CDO1* DNA from DLD1 detected methylation in PCR reactions using 100, 10, or 1 ng/reaction, whereas it could barely detect methylation in PCR reactions using 0.1 or 0.01 ng/reaction. No methylation was detected at any concentration of DNA in reactions using the HepG2 negative control DNA (Fig. 1d). These findings suggested that even dense *CDO1* methylation such as that found in DLD1 cells can barely be detected using Q-MSP when the DNA is diluted about 1000 fold, and that detection is less sensitive when less DNA is applied. Therefore, in order to detect minimally residual disease of cancer in body fluids, it would be better to be able to use a higher volume of DNA than that usually used for quantitative analysis of DNA methylation.

### Detection of methylation of the CpG island sequence of the *CDO1* gene in the plasma of CRC patients by Q-MSP using a low amount of template DNA

**Figure 2:**
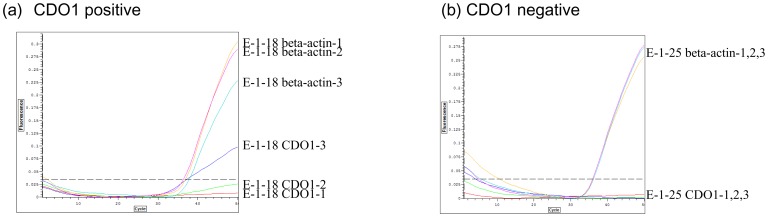
Q-MSP analysis of methylated *CDO1* in plasma using a low volume of template DNA (termed one template volume). (**a**) A representative positive case was shown. The β-actin gene was assayed as an internal control for bisulfite treated DNA. Even for this positive case, detection of methylated *CDO1* is not stable, because all the templates did not overcome the threshold level. (**b**) A representative negative case is shown. Note that the β-actin level is lower than expected.

We then examined the sensitivity of detection of *CDO1* methylated DNA in the plasma of 20 CRC patients. In this preliminary experiment, we extracted DNA from 400 µl of CRC patients plasma. Half of the extracted DNA was applied to triplicate assays of both *CDO1* and β-actin. Thus the plasma volume for each individual assay was 33.3 µl, which designated as 1 template volume. *CDO1* methylation was detected in only 2 (10%) of the 20 CRC patients, while β-actin methylation was detected in all samples (Fig. 2). Fig. 2a shows a *CDO1* positive cases; methylation of *CDO1*-3 was clearly detected, but that of *CDO1*-1 and *CDO1*-2 was marginal, because the signals were below threshold. However, this level of methylation may be valid, as PCR of 1/1000 dilution of the positive control exhibited a similar signal (Fig. 1d). In contrast, *CDO1* negative cases never exhibited any signal at all of amplified *CDO1* methylation (Fig. 2b). For these experiments, the amounts of DNA extracted from the initial 400 µl of plasma averaged 435 ng, ranging from 160 ng to 2000 ng. Therefore the amount of DNA applied to 1 PCR reaction corresponded to at least 16.7 (1/6) ng/reaction. This amount of DNA is thought to be sufficient for Q-MSP, if methylated alleles are included (see Fig. 1c). However, the Ct value for β-actin ranged from 34 to 38, which was totally different to that obtained when performing Q-PCR of cell lines, in which the usual Ct value of β-actin is around 28. This result suggested that detection of plasma DNA was less sensitive than that of DNA from cell lines, likely due to DNA degradation or other unknown reasons. Sufficient DNA for the PCR reaction was present only in 1 of 2^6^ (64 fold) to 2^10^ (1024 fold). This meant that the initial plasma volume used for DNA extraction should ideally be increased 100 to 1000 fold (i.e. to 4–40 ml plasma) for assay of plasma DNA using Q-MSP. Therefore, to obtain enough plasma DNA to detect minimal residual disease, more DNA than that used in this preliminary study would be needed. Thus our initial preliminary study had not used optimal conditions for detection of *CDO1* methylation in plasma.

### Detection of the methylated CpG island sequence of the *CDO1* gene by Q-MSP using increased amounts of DNA from the plasma of CRC patients

**Figure 3:**
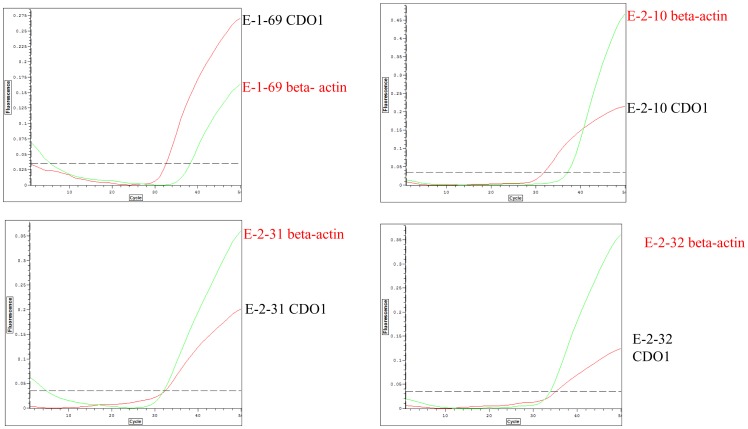
Increasing the amount of plasma used for DNA extraction (to three template volumes) for Q-MSP analysis of methylated *CDO1* in plasma enhances the signal. (**a**) E-1-69 (**b**) E-2-10 (**c**) E-2-31 (**d**) E-2-32 that were positive for methylation of *CDO1* in plasma when the template DNA was extracted from a higher volume of plasma.

We then examined DNA extracted from the equivalent of 3 template volumes for detection of *CDO1* methylation in the plasma of the 20 CRC patients. This assay detected 4 cases (20%) that were positive for methylation of *CDO1* in plasma. Moreover clearer signals were obtained than when one template volume was used, indicating that the sensitivity of the assay may be greatly augmented by increasing the amount of the plasma-derived DNA (Fig. 3).

**Figure 4:**
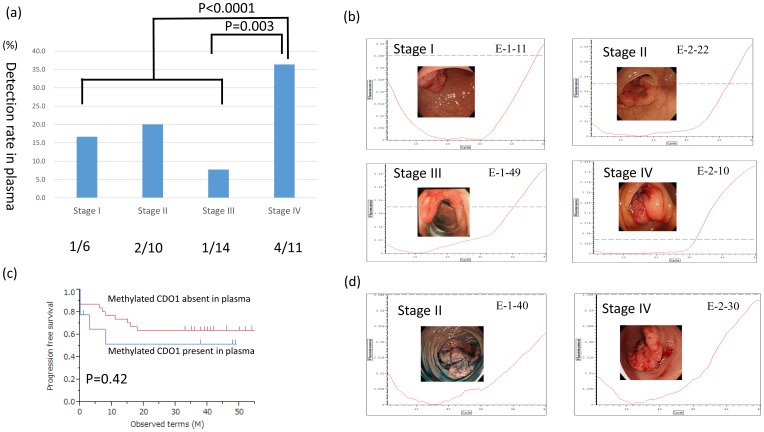
Detection rate of methylated CDO1 in the plasma of patients with CRC using a high volume of plasma-derived DNA template. (**a**) Q-MSP analysis of methylated *CDO1* using a high amount of plasma-derived DNA of CRC patients at differential stages. (**b**) Representative positive cases are shown according to stage. A picture of colon fiber in the primary cancer is included in each figure. (**c**) Survival curve of CRC patients according to methylated *CDO1* levels in the plasma. (**d**) Representative cases showing marginal positive case are shown.

We then examined 40 independent CRC cases by Q-MSP using DNA extracted from a larger volume of plasma (1.0 to 2.5 ml plasma corresponding to 5 to 12.5 template volumes based on our initial template volume definition). The average amount of DNA extracted DNA was 1491 ng, (ranging from 580 ng to 2590 ng). This assay detected clear *CDO1* methylation in 8 the 40 CRC cases (20%). Clear methylation was detected in 1 of 6 (16.7%), 2 of 10 (20.0%), 1 of 13 (7.7%), and 4 of 11 (36.4%) of stage I, II, III, and IV CRC cases, respectively (Fig. 4a). The higher the stage, the higher the methylation level that was detected (Fig. 4b). Patients' backgrounds are shown in Table 1. Although *CDO1* methylation is more frequently found in CRC patients with distant metastasis, the prognosis of patients with methylated *CDO1* in plasma was not significantly worse than patients in which no methylated *CDO1* was detected in plasma (Fig. 4c). Below threshold signal corresponded to 1/1000 dilution of the positive control (Fig. 4d). If such a marginal below threshold signal of *CDO1* methylation was included as a positive signal, then methylation was detected in all 40 of the CRC patients.

### Combination of methylated *CDO1* with increased serum CEA/CA19-9 for detection of curable stage I to III colorectal cancer

**Figure 5:**
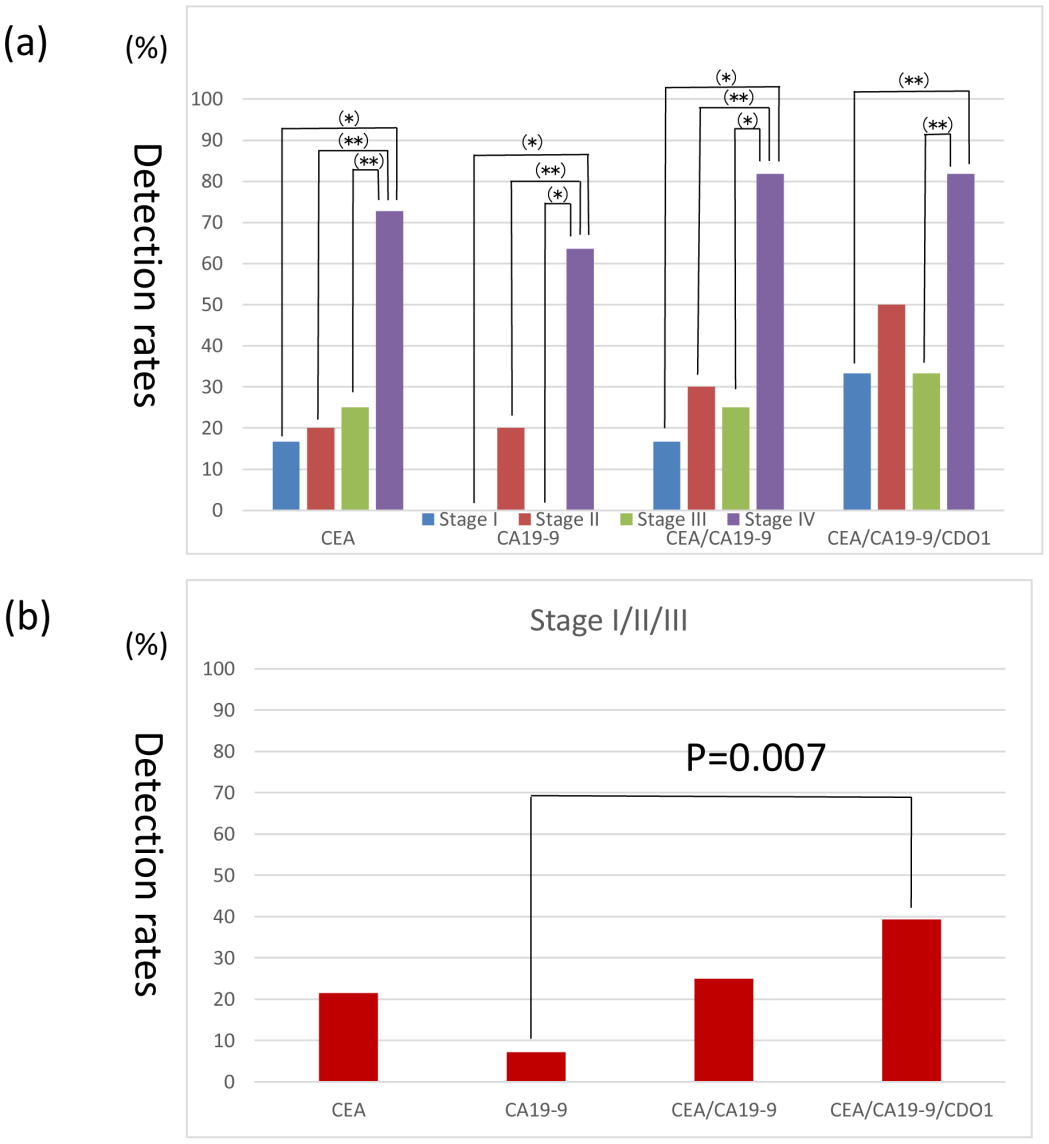
CRC detection rate of CEA, CA19-9, and combinations of the tumor markers. (**a**) CRC detection rate of CEA, CA19-9, combination of CEA/CA19-9, and combination of CEA/CA19-9/plasma *CDO1* methylation according to CRC stage. (*) indicates p<0.01. (**) indicates p<0.05. (**b**) Curable CRC (stage I to III) detection rate was increased by combining plasma *CDO1* methylation data.

Of the above 40 patients, 39 were informative regarding the preoperative value of serum CEA and CA19-9. CEA was positive (>5.0 ng/ml) in 14 of these 39 patients (35.9%), while CA19-9 was positive (>37.0 IU/ml) in 9 of these 39 patients (23.1%). The rate of detection of CRC according to stage was calculated with respect to CEA, CA19-9, the CEA/CA19-9 combination, or the CEA/CA19-9/plasma *CDO1* methylation combination (Fig. 5a). The rate of detection of stage IV CRC by CEA, CA19-9, or CEA/CA19-9 was high. However combining plasma *CDO1* methylation data with CEA/CA19-9 did not increase the detection rates. On the other hand, *CDO1* increase the detection rates of Stage I, II, and III CRC, which are regarded as curable (Fig. 5a). Although only 21% of such curable patients could be detected based on CEA levels, *CDO1* combination increased the detection rate of such curable patients to 39.3% (Fig. 5b).

## Discussion

Since it was discovered that cancer-specific alterations in blood can be detected in cancer patients, numerous tumor markers have been developed for clinical application. In CRC, serum CEA and CA19-9 are well recognized as such useful markers [18], [19]. Since high values of serum CEA and CA19-9 are frequently detected in stage IV colorectal cancer, these markers are useful for the prediction of prognosis or for case of early detection of recurrence in the outpatient center [18]. Because these markers can sometimes detect recurrence earlier than diagnostic imaging such as CT or echo, they are indispensable for physicians who treat CRC as a clinical tool. On the other hand, unfortunately these markers are not sufficient to detect cancer when applied to the detection of curable CRC. The pre-operative CRC detection rates of CEA and CA19-9 are about 30∼50% or Stage I CRC and 10∼20% for stage III CRC [20]. If a novel tumor marker in blood could be used in combination with such classical tumor markers, this would increase the detection rate of curable CRC and these markers would show promise for the purpose of screening CRC in medical examination [21].

Alterations that are to be used as tumor markers must be cancer specific. The methylation of the *CDO1* promoter region that was assayed in the current study was originally identified by robust screening of a pharmacological unmasking microarray that was carried out to explore cancer-prone methylation[1], [2], [9]. By using such an array we have identified numerous novel genes with cancer-prone methylation. *NMDAR2A* [6], *DFNA5* [7], *OSMR* [8], and *CDO1* [9]. Of these genes, *CDO1* stood out with regard to sensitivity and specificity for the differential detection of CRC from the corresponding normal mucosa. Similar *CDO1* methylation traits were confirmed in various other cancers such as breast, esophageal, lung, bladder, and gastric cancer. Because such cancer-prone methylation is rare, analysis of *CDO1* methylation has great clinical potential for detection of minimal residual disease in human body fluids such as blood.

*SEPT9* is considered to be the first molecule in which frequent methylation was successfully detected in the plasma of CRC patients by using real time PCR [16], [17]. The detection rate of methylated *SEPT9* in the plasma of curable CRC patients with Stage I to III is remarkably high, being over 60% [22]. Intriguingly, such a high detection rate of a methylated DNA in the plasma of patients with curable CRC was also shown by using another method. Thus, by using the methyl BEAMing method, Li et al. demonstrated that plasma methylated *Vimentin* could be detected in the about 40–60% of curable CRC at stage I to III, and that the sensitivity of detection was higher than that of the serum value of CEA [21]. Based on these results, analysis of methylated DNA is a very promising candidate tool for blood diagnosis of cancer, and plasma *CDO1* methylation that was the focus of this study considered as one such candidate DNA. Cancer specificity and sensitivity of *CDO1* methyaltion was proven to be equal to those of either *SEPT9* or *Vimentin* methylation in CRC, where the AUC of the ROC curve to differentiate cancer tissues from the corresponding normal mucosa was 0.96 [9].

In contrast to these promising reports, there have been some disappointing reports with regard to the detection rates of methylated genes in plasma [14]. The detection rate of methylated *CDO1* in plasma is a disappointing 20% of the total CRC patients assayed, and is about 35% in stage IV CRC. In our current study, we made DNA templates of the *CDO1* promoter of DLD1 cells, positive control for *CDO1* methylation, diluted the DNA up to 100,000 fold, and compared the *CDO1* methylation detection level of different dilutions. This assay showed that the smaller the amount of DNA template that is applied, the poorer is the efficacy of PCR detection. Allowing for -actin amplification efficacy, the amount of DNA in the total DNA content of plasma that could function as a template for Q-MSP PCR amplification *of CDO1*” was unexpectedly extremely small (the efficacy was calculated as 1/100 to 1/1000). In our current study, the sensitivity of methylated *CDO1* by conventional Q-MSP is above 1/1000 dilution of the extracted DNA (Fig. 1d), which is much inferior to that of the BEAMing assay (which showed 1/10,000 dilution sensitivity) [23]. When the volume of plasma used for DNA extraction was increased by 3X (defined as three template volumes), clearer signal was obtained in Q-MSP. However, increasing the plasma volume used for DNA extraction by 10X (ten template volumes) did not provide as good a detection rate as that obtained using 3 template volume. Since 10 to 12 template volumes were used for Q-MSP PCR amplification of methylated *SEPT9* [16], the number of DNA templates of our study were not so small as those used in the SEPT9 study. However methylated DNA detection rates were much lower in the present study than in the *SEPT9* study, since methylated *SEPT9* was detected in around 60% of curable CRC [16].

Due to the low sensitivity for the assay (20%) of plasma methylated *CDO1*, the utility of the test for population screening for CRC will require improved sensitivity for detection of early cancers and advanced adenomas. There may be ways to achieve possible improvements as follows; 1) 3.5 to 5 ml of plasma should be collected for DNA extraction. 2) CDO1 PCR primer concentrations were increased. 4) A subject was positive if any of the three PCR replicates were positive, because a third PCR measurement necessarily increases sensitivity and decreases specificity. Our next challenge would be reached by such devises to improve sensitivity for detection of minute cancer cells. If we can increase the sensitivity by the above devises, we will collect the plasma from healthy persons and can determine the most optimal cut-off value of methylated CDO1 in CRC patients.

There are 2 possible explanations for the fact that the detection rate of *CDO1* methylation were much inferior to those of *SEPT9* methylation in the plasma of CRC patients. Thus, the detection rate as determined using Q-MSP could be elevated by reducing the threshold level. For example, in Fig. 4d, this stage IV was judged as negative for *CDO1* methylation. However, the threshold was reduced, this case could be considered as positive. It is possible that the threshold can be further reduced. Although we didn't assay the plasma of a healthy person, the threshold should be determined in comparison to the detection rate of a healthy control as previously shown [16]. The second explanation is that the level of methylated *CDO1* in plasma is less than that of methylated *SEPT9*. CDO1 is involved in cysteine metabolism, and cysteine biology has recently gained attention in terms of cancer stem cell theory. The cysteine transporter xCT is associated with, the cancer stem cell marker CD44, and with an increased cysteine concentration in the cytoplasm of cancer stem cells, resulting in inhibition of ROS generation and chemoresistance. Cancer stem cells are believed to survive longer in primary cancer tissues, suggesting that less DNA may be derived from cancer stem cells than from otherwise cells. If the latter theory is correct, methylated *CDO1* is not appropriate for use as a plasma tumor marker. If the former theory is correct, detection rates of cancer cells based on plasma methylated *CDO1* could be increased by improvement of the detection system. In our current study, the amount of DNA that is useful for such detection was shown to be low in plasma. Therefore, the use of nested PCR is a promising approach for such analysis in the near future.

In conclusion, analysis of methylated *CDO1* in plasma is disappointing with regard to detection rate. However plasma methylated *CDO1* is independent of serum CEA/CA19-9, so the combination of these markers could increase the detection rate of curable CRC. On the other hand, the CRC detection rate was not increased by combination of plasma methylated *CDO1* data with CEA/CA19-9 levels in Stage IV CRC. Therefore, if the CRC detection rate based on methylated *CDO1* in plasma were to be increased by using the novel detection system described above, plasma methylated *CDO1* analysis would be a promising tool for detection of curable CRC by medical examination of blood. Moreover, since *CDO1* methylation is universally found in other cancers, this technique may be a broadly applicable method for cancer detection by using blood.

## Supporting Information

Table S1 PCR production and sequence of primers and fluorescent probe.
(XLSX)
